# A Methodology for Choosing Time Synchronization Strategies for Wireless IoT Networks

**DOI:** 10.3390/s19163476

**Published:** 2019-08-09

**Authors:** Francisco Tirado-Andrés, Alba Rozas, Alvaro Araujo

**Affiliations:** B105 Electronic Systems Lab, ETSI Telecomunicación, Universidad Politécnica de Madrid, Avda. Complutense 30, 28040 Madrid, Spain

**Keywords:** methodology, time synchronization, IoT

## Abstract

The wireless Internet of Things (IoT) family grows without interruption. Every day more applications and wireless devices are available to interconnect and help solve multiple problems in areas such as health, critical infrastructure, industry, etc. Many of the tasks to be performed by the IoT network require time synchronization for their correct operation, either to use the spectrum more efficiently, to add data from different sensors, or to carry out coordinated communications. Each of these applications has different requirements regarding time synchronization. This means that the decision of which strategy to follow to synchronize an IoT end device becomes a task that requires important prior analysis and usually, if developers are experts in the topic, ends with the implementation of an ad hoc solution. In this article, we present a methodology to choose an adequate time-synchronization strategy for any wireless IoT application. We also present a tool that executes the methodology, guiding the IoT application developer through some input forms. This combination of methodology and tool abstracts developers from the complexities of time-synchronization strategies, allowing them to choose the correct strategy regardless of their level of knowledge in wireless IoT time synchronization. As a result, the methodology offers a set of time-synchronization strategies that are adjusted to the needs of developers and applications.

## 1. Introduction

The Internet of Things (IoT) is the integration of connectable devices and sensors to enable remote monitoring, creating a network of networks of autonomous objects. The wireless IoT family is expanding into different areas such as vehicles, buildings, construction equipment, smart wearables, smart home automation, smart health monitoring, smart cities, smart security, etc. Every day more applications and wireless devices are available to interconnect and solve multiple and diverse problems. According to the Gartner Inc. 2017 prediction, the IoT will include 20.4 billion units installed by 2020 [1] with almost $3 trillion of investment. The numbers vary from source to source, but it is agreed by everyone that the number of IoT connected devices installed worldwide will continue increasing [2,3] and that in the near future IoT devices are expected to be integrated in a larger number of areas (home, industrial, medical, etc.) and applications (smart security solutions, smart home automation, smart health care, smart wearables, transportation, building automation, renewable energy, etc.). The IoT is the cornerstone of many organizations’ digital transformation, enabling them to optimize existing operations and excel at creating and pursuing exciting new business models [4]. The traditional fields of embedded systems and wireless sensor networks (WSNs), among others, all contribute to enabling the IoT.

Wireless IoT networks are composed of autonomous sensor nodes that are spatially distributed to monitor physical or environmental conditions, and share their data through the network via wireless communications. One of the key elements, in most of the main applications developed on IoT, is time synchronization. Time synchronization, or the way an IoT device adjusts its internal clock in order to align with the clocks of other devices in the network is crucial because of wireless communications. An adequate time-synchronization strategy allows the network to achieve a reliable, energy-efficient and low-latency communication. Another reason to consider time synchronization a vital characteristic in IoT networks is that a coordination of events in different nodes is possible with it. Therefore, information from different nodes can be merged to obtain a complete picture of the scenario under monitoring. Synchronization is also at the center of many of today’s IoT challenges, particularly for low-power IoT networks and end devices.

Synchronizing an IoT network is not a trivial task. Clocks drift out of synchronization, especially those using low cost, commodity computing parts that are often used in low power IoT. Since each of the devices in the network has its own clock, a synchronization strategy is needed to make them all work in unison. The most common strategy consists in synchronizing all the devices’ clocks with a reference clock. In most cases it is not enough to synchronize local clocks once at the beginning of the application because, usually, local clocks in the wireless devices are not stable enough to keep the synchronization in the network during the lifetime of the application. In most cases, more advanced synchronization strategies are needed to keep the application properly synchronized during its whole operation.

There are many factors that affect time synchronization causing dynamic deviations over time, such as temperature, oscillator aging, battery charge, pressure, etc. [5]. For example, temperature changes affect the accuracy of local clocks by dynamically modifying their frequency and causing them to deviate from the reference. Other factors that affect an accurate and stable time synchronization are the number of nodes in the network, the topology, the type of message distribution, software layers with non-deterministic delays in communication processes, attacks from malicious time sources, etc. Some of the practical aspects that affect time synchronization in wireless IoT networks can be seen in [6].

There is a wide variety of IoT applications. Due to that variety, time-synchronization requirements in IoT networks are very different, going from very lax to very demanding. Every time developers face the problem of choosing and implementing a time-synchronization strategy for a specific wireless IoT application, they realize that a huge research is necessary before undertaking the task. Solving the time-synchronization challenge must be undertaken taking into account many other trade-offs, because time synchronization is just another service in the IoT application routine. There is no single time synchronization strategy that maximizes all parameters, nor is there a synchronization strategy that works for every application. For example, in most cases, the more precise the time synchronization strategy, the more message exchanges between nodes that are needed, or the better and more expensive hardware it requires [7]. Strategies can pursue an accurate time synchronization in top layers, such as the application layer [8], or in low layers, such as the medium access control (MAC) layer [9].

Due to the usefulness of IoT networks in multiple fields, the applications based on wireless IoT end devices have diversified and expanded. Nowadays, it is possible to find wireless network applications dedicated to monitor parameters in a distributed way [10]. Some wireless networks coordinate sleep wake-up node scheduling mechanisms [11]. There are also some wireless network applications with communication protocols based on time slots to transmit and receive messages making an efficient use of the radio spectrum [12]. All of them need time-synchronization strategies to operate correctly. Each of these applications has different needs with respect to the time synchronization of the network. Thus, many strategies to synchronize wireless IoT networks have appeared over the years and continue to do so, adapting the solutions to a specific problem.

Clock values of different IoT end devices diverge from each other and, consequently, many clock synchronization protocols for wireless networks have been proposed in the past. These protocols differ from each other considerably due to their structural features, technical features, or global objective features [13]. To synchronize a wireless network, it is necessary to reach a common notion of time taking into account that the clocks of the devices can deviate due to multiple reasons, that the time requirements for every application vary, and that the target parameters to maximize in the application differ in every case. For example, there are single-hop vs. multi-hop wireless networks, internal vs. external clock synchronization, stationary vs. mobile nodes, fault tolerance, scalability, sleep scheduling, energy efficiency, etc. The approach of every clock synchronization protocol is different due to the limited resources of every network, the nature of the communication or the physical environment. To develop an appropriate time-synchronization strategy several things need to be known: the source from where the clock value is propagated, how the clock value is propagated, when the clock value is propagated, how the logical clock time is determined from the physical clock value, if the time synchronization is a priori or post facto, or if the synchronization is probabilistic or deterministic, among other concerns.

Due to the complexity and the challenges involved in choosing the synchronization strategy in each case, a methodology for choosing time-synchronization strategies for wireless IoT networks is presented in this work. Developers can focus their efforts in the application and main services because the methodology abstracts them from the complexity of the time-synchronization strategies, facilitating their choice and assuring a strategy adapted to the IoT network needs. The methodology helps experts and developers who lack the experience in time synchronization to find the appropriate solution for specific cases.

The remainder of this article is structured as follows. In Section 2 a brief discussion of some related work is presented. We introduce our methodology proposal in Section 3. Section 4 presents a tool to execute the methodology. Section 5 describes several use cases to justify the utility of our methodology. We conclude our work in Section 6.

## 2. Related Work

A model for the classification of synchronization protocols in wireless networks can be found in [13]. Through this survey, it is possible to understand the difficulty of choosing the right synchronization strategy adapted to a particular application. The authors make a classification dividing the synchronization protocols into three main groups: structural features, technical features and global objective features. The authors also classify several representative clock synchronization protocols into those categories.

To face the time-synchronization challenge we can find different approaches. The hardware approach is based on using additional hardware modules to improve some characteristics of the network to enhance some parameters, for example, to make the strategy more accurate or more stable. For example, in [14] an innovative wireless end device is presented to monitor structures in a synchronized way. In [15] the researchers make use of the Global Positioning System (GPS) and in [16] the networks base their time synchronization on real-time clock (RTC) modules.

The software approach centers its efforts in the development of software algorithms and strategies to improve the time-synchronization techniques. Some software proposals try to correct the drift of the clock by compensating the temperature variations [17]. Some software approaches highlight the critical and challenging security issue in the IoT time-synchronization process [18]. Some of those synchronization protocols have become reference protocols, such as Reference Broadcast Synchronization (RBS) where the broadcast nature of the physical channel is exploited to synchronize a set of receivers [19], the Timing-sync Protocol for Sensor Networks (TPSN) which is based on a tree topology [20], or the Maximum Time Synchronization (TMS) where the idea is to maximize the local information to achieve a global synchronization [21].

The objectives of time-synchronization strategies are very diverse and, therefore, synchronization strategies can be applied in very different ways. Among all the synchronization protocols, there are some that need specific network topologies, such as a tree topology like the Flooding Time Synchronization Protocol (FTSP) [22]. Some need one-way message exchanges, others instead, need two-way communication [23]. Some do receiver-receiver synchronization while others synchronize the sender with the receiver(s) [24].

Whenever developers are faced with the decision to choose a synchronization strategy that suits the needs and requirements of their application, they realize that they have two options: develop their own strategy or adapt another strategy to their own needs. Developers can base their decision on published works. There are many published surveys, such as [25], where the need for synchronization in wireless networks is raised; [26], where the authors focus on a statistical signal processing framework; [27], where a set of protocols for decentralized wireless networks is presented; or [28], where different existing methods for time synchronization are studied. However, today, no methodology is found among the published scientific documentation that helps developers find the most appropriate solution for each case.

As mentioned earlier, the IoT family of end devices continues to grow and is becoming more varied. Therefore, the task of synchronizing a greater quantity and variety of end devices becomes more complex every day. Similarly, since the task of synchronizing a wireless network is becoming increasingly complex, the number of experts in the field is reduced. Therefore it is necessary to create and use a methodology to address this task of selecting time-synchronization strategies appropriate to each scenario and wireless network. Nowadays, there is a lack of methodologies to assess these time-synchronization strategies, to compare them, and to determine those that are best suited for a given network, scenario and application.

## 3. Proposed Methodology

The methodology presented in this article offers IoT developers a way to choose a time-synchronization strategy in a methodical way. The proposed method provides developers, looking for a synchronization strategy for their IoT network, with a set of strategies that fit the particular needs of each case. In this way, developers do not need to be experts in the particular issue of time synchronization in order to choose the correct strategy. Each synchronization strategy has been previously evaluated by an expert in the field and has been cataloged based on multiple parameters. Thanks to this taxonomy, depending on the parameters that are important for each application and developer, the methodology is able to generate a solution or a group of solutions adapted to the particular case. It is ensured that, regardless of the level of expertise of the developers that make use of the methodology, the solution that is finally chosen has been technically validated. The methodology compensates for the lack of experience that developers may have and ensures a successful decision.

**Figure 1:**
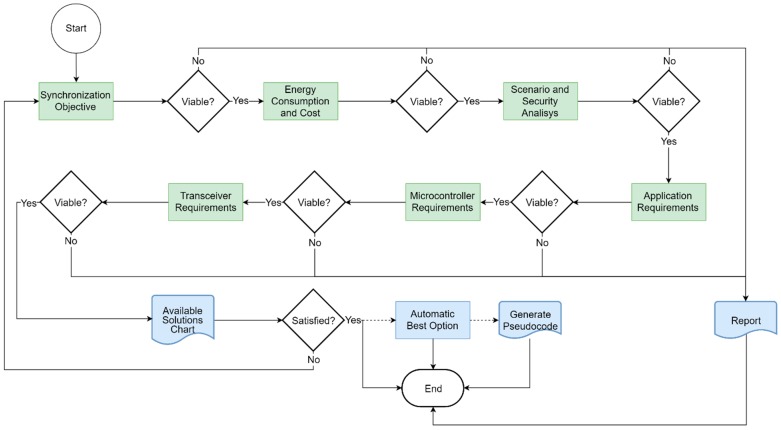
Flowchart of the proposed methodology where the input blocks are colored in green and the output blocks are filled in blue color. White blocks are those where the methodology makes decisions.

The methodology we propose is based on a series of steps that can be observed in Figure 1. Each of the steps, which we will call blocks, evaluates a series of characteristics or parameters. All synchronization strategies are available at the beginning of the cycle. Developers select certain characteristics or requirements in each of the blocks. Since not all the strategies comply with the requirements imposed in each of the blocks, the number of available strategies is reduced as one advances through the blocks. Once the entire process is completed, all the remaining time-synchronization strategies meet the commitments established between the requirements and the available resources. We call this procedure “filtering the time-synchronization strategies”. This filtering process makes necessary a selection of time-synchronization strategies among all the possibilities in order to find a time-synchronization strategy adapted to the needs of the application and the developer.

This methodology is dynamic and can handle the appearance of new solutions such as new synchronization protocols or situations, or changes in the needs or in the environment.

The methodology guides the developer through a sequence of topics related to time synchronization from the most abstract to the most concrete. In this way, the methodology can filter through multiple interrelated parameters, according to the interests of the developers, selecting the most appropriate synchronization strategy in each case.

The methodology does not only automate the selection of a synchronization algorithm, but it is able to offer non-expert developers in the field of time-synchronization solutions adapted to their needs. If the needs of the application change, this methodological process is carried out again and a new adapted solution is obtained. An ad hoc solution would always be a more fitted solution, but most IoT application developers are not experts in the field of time synchronization, and the development of an ad hoc synchronization protocol would take a lot of effort on their part.

In this article, every block of the methodology is explained. This article also provides some tools that show how the evaluation should be undertaken. The objective of our methodology is not to specify what should be done in the evaluation, but rather how to do it. However, in this article we also present a set of tools that make the execution of the methodology possible.

The methodology we propose can be represented by a flowchart as shown in Figure 1. This flowchart can be divided in six main blocks of inputs (colored in green) plus the output block (colored in blue). The white blocks represent the phases where the methodology makes decisions, that is, where the methodology compares the input requirements with the available time-synchronization strategies and filters them.

The flow of the methodology shows, one input block after another, how the possibility of finding a viable solution is evaluated. If a possible solution is not found given the requirements, a report is generated. This report tells the developers what are the parameters, or trade-offs between them, that make a viable solution impossible. The cycle ends after determining its non-feasibility. By contrast, if all the input blocks are evaluated, and there are still viable solutions available, a chart is generated. This chart shows the relationship between the parameters and the possible solutions that meet the requirements that the developers have introduced. If the developers are not satisfied with the solutions offered by the methodology, they can always start the process again and modify the inputs.

The order of the input blocks, as shown in Figure 1, can be interchanged. The established order ensures a method of filtering the time-synchronization strategies from the most abstract to the most concrete characteristics. The first block of inputs in the methodology evaluates the objectives of the time-synchronization strategies. We call that block the “synchronization objective” block. We have chosen three of the most common objectives that are pursued when developers want to synchronize an IoT network. Most synchronization strategies belong to one of these objectives: spectral or energy efficiency making use of MAC schemes, data fusion, or communications with cooperative transmissions. The second input block is the “energy consumption and cost” block. In this block the methodology evaluates the developer’s interest in the additional cost (monetary or in resources) and the developer’s interest in the energy consumption of those tasks related to time synchronization. The third block evaluates the scenario where the IoT network is going to be deployed. This block is called “scenario and security analysis” because it is here where the methodology evaluates if the scenario facilitates or hinders the time-synchronization tasks and how critical is the security and the authenticity of the time source. The variation of the environmental conditions, especially changes in temperature, can affect the behavior of the clock sources to a greater or lesser extent. Time-synchronization strategies can be different if the network is oriented to indoor spaces, or if it is a network that is planned to be installed in open and outdoor spaces. As previously mentioned, each application may have specific requirements that need to be known. The block that evaluates those specific “application requirements” is called with that same name. In this block the time accuracy needed for the synchronization strategy for a proper execution of the application is evaluated. The maximum time-synchronization error allowed is a key factor for the correct operation of the application. This is one of the most important parameters in terms of synchronization strategies. It sets the error limit that the network can tolerate and, if it is exceeded, that time-synchronization strategy is considered to be not valid. In this block other concepts such as the network size or the convergence time are also evaluated. The network size is the number of nodes that must be synchronized in the network. The greater the number of nodes, the more complex and/or slow the message distribution is likely to be. The synchronization convergence time is the time the strategy needs from the start of its execution until it reaches the synchronization objective. This convergence time can be affected by the network size. Certain synchronization strategies run correctly for a small number of nodes, but if the number of nodes in the network grows, the accumulated error also does.

The last two blocks of inputs evaluate the microcontroller and the transceiver hardware resources. The tools used to execute the methodology can filter the available synchronization strategies based on the hardware limitations or available resources. The number of time-synchronization strategies is reduced if those strategies must be implemented on a specific hardware platform. If there is no specific hardware platform to implement the strategy, the methodology assumes that there is freedom to choose the platform that best suits the application needs. In the same way, if a radio transceiver is already selected, or if the topology of the network or the distribution of the messages is already imposed by the requirements of the application, the number of synchronization strategies that can be implemented is reduced.

Any of the input blocks can be skipped, considering in that case that there are no limitations or, in other words, that there is freedom to choose any available option.

**Figure 2:**
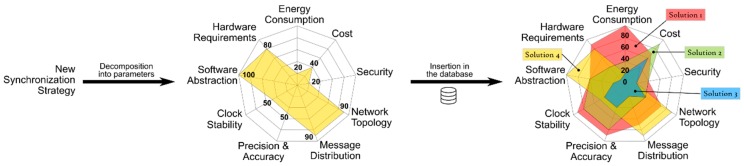
Graphical representation of the insertion of a new synchronization strategy into the database with all the possible solutions. This example shows the insertion of the fourth solution.

Once all the input blocks have been completed a radar chart is generated. This radar chart is a graphical method of displaying nine parameters that characterize a synchronization strategy in the form of a two-dimensional chart. Each of the parameters have a quantitative value, from 0 to 100, and that value is represented on axes starting from the same point at the center, as can be seen in Figure 2. As a general rule, each synchronization strategy has just one main “synchronization objective”. For each objective a set of solutions can be considered as valid. The rest of the input blocks of Figure 1 are represented by the nine radar parameters. The energy consumption and the cost are related to the second input block, the security, the topology of the network and the message distribution represent the “analysis of the scenario and security”, and the “requirement of the application” is represented by the precision and accuracy, the stability of the clock and the abstraction of software. The last two input blocks are included in the hardware requirements. All the available time-synchronization strategies have particular values for each of the evaluated parameters. All the strategies that can be plotted whose area stays inside the area of an available solution are offered to the developers as the synchronization strategies that meet the IoT network requirements. When developers select any of the solutions proposed by the radar chart the methodological cycle is considered finished. In certain cases, the tools that execute the methodology are able to propose what they consider the best option among all the proposals. If developers agree with this solution, the methodology cycle finishes. Finally, in special cases, a pseudocode is offered to facilitate the implementation task of the strategy in the IoT network. The methodology does not define time-synchronization protocols, it catalogs them and offers the ones that are adjusted to the requirements of a specific application.

Through this methodology, we can standardize, structure, and organize the way in which time-synchronization strategies are filtered and selected based on the application and the developer decisions or needs. This methodology is repeatable, reproducible, and reports results in a clear way. The methodology helps to tackle different projects in the same way and allows any developer to select the appropriate time-synchronization strategy for his or her wireless IoT network, regardless of his or her experience in the subject.

## 4. Proposed Tools

A series of tools have been created to go through this methodology allowing the evaluation and the introduction of the necessary data in the input blocks. Two web forms and a database have been developed. One of the forms is used by experts in time-synchronization strategies to introduce each of the strategies in a parametrized way into the database. The other form is the one that the application developers would use to introduce their requirements regarding synchronization and to make the queries to the database to finally obtain the radar chart.

The input form for experts consists of multiple fields that ask for each of the parameters and input blocks of Figure 1. The process to incorporate a new synchronization strategy to the system is represented in Figure 2. The methodology does not change, the database of possible solutions simply grows with this new incorporation. The new strategy should be analyzed by the experts and broken down and cataloged according to the entry form. Each of the nine parameters of the radar chart take a value. The values go from 0 to 100, in relation to the quantity of resources needed to fulfil the parameter demand. In other words, low values mean more freedom in the selection, fewer requirements, less exactitude, and consequently a smaller area in the figure. But high values mean more rigorousness, more demands, more requirements, and a greater area in the figure. Once incorporated into the database, this new strategy is part of the possible solutions that are offered to developers who make use of the methodology and the selection tool.

The expert form—where synchronization strategies are introduced—has multiple fields which will be briefly described next. The first section of the form asks about data such as the name of the strategy, if it has related publications, the year of publication, etc. Then, the form asks about the nine points from which the radar chart is created. In some of these points there are some detailed questions, such as the number of clocks in the system, the type of clocks, the radio frequency bands, whether software modifications can be made in the MAC layer or only in the application layer, etc. In addition, the form also wonders if the system has some type of clock correction, whether the offset and/or the skew are dynamically corrected, if these corrections are deterministic, probabilistic, etc. Finally, the form asks if there is any type of pseudocode associated with the strategy.

This article is focused on the form for developers to make the queries to the database. The most direct and effective way to know the requirements and needs of the application regarding time-synchronization is to ask the developers. That is why the form used to obtain the necessary inputs is structured as several blocks of questions. We have classified these questions according to their impact, therefore creating a priority hierarchy.

**Figure 3:**
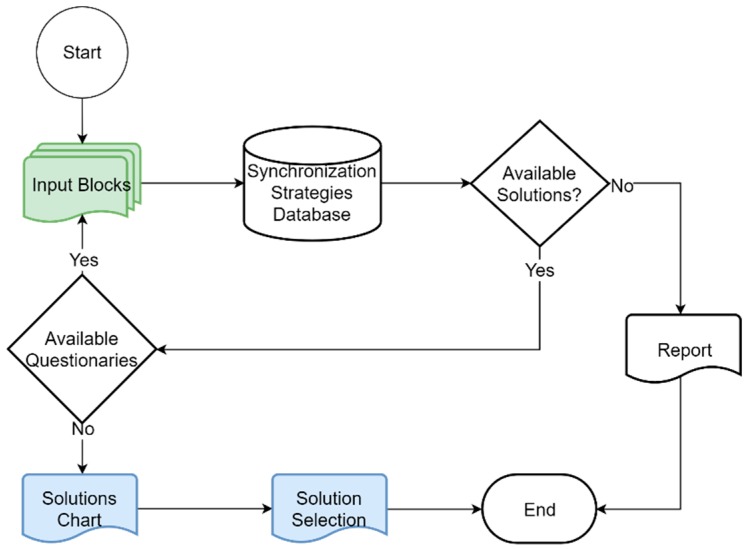
Flowchart of tools where all the input blocks are evaluated and queries to the time-synchronization strategies database are generated. The cycle ends when there is no solution available to fulfill the requirements or when all the input blocks are evaluated and the radar chart is generated.

There are open questions, closed questions and questions about the specifications and requirements. Open questions are more general than the closed questions, which are more specific. The input form follows the flowchart of inputs of the methodology. In Figure 3 the flowchart followed by this input tool is shown. Each of the topics of the input blocks is evaluated. The web form starts with the first input block, the “synchronization objective” block. Several questions, open and closed, are presented to the developer. Once the developers move forward their answers are evaluated and a query is made to the database. If after the evaluation there are still available solutions another input block is processed. If there are no remaining input blocks, the radar chart is generated, and the solution is chosen before the cycle ends. If there are no available synchronization strategies to fulfill the requirements after all queries are made to the database, a report is generated, and the cycle ends.

The input forms contain general questions and detailed questions. Depending on the answers of the general questions some of the detailed questions can be considered irrelevant. The more information obtained from these input forms, the more accurate the result. The more details are provided, the narrower the selection filter of time-synchronization strategies. The narrower the filter, the fewer the strategies that appear as available. Different developers may have a relative interpretation of the same metric, for example, the accuracy of an oscillator. It is the tool who is in charge of translating the relative to the absolute.

There is a direct relation between the requirements and the number of available solutions. If, on the one hand, due to the requirements introduced in the input blocks, the filter is too selective and restrictive (bigger area in the chart), it would be possible that no solutions meet all the desired parameters. If, on the other hand, few details are provided, the selection filter is too flexible and tolerant (smaller area in the chart) and, therefore, a large number of possible solutions are offered, many of which are very general and poorly adjusted to a specific problem.

**Figure 4:**
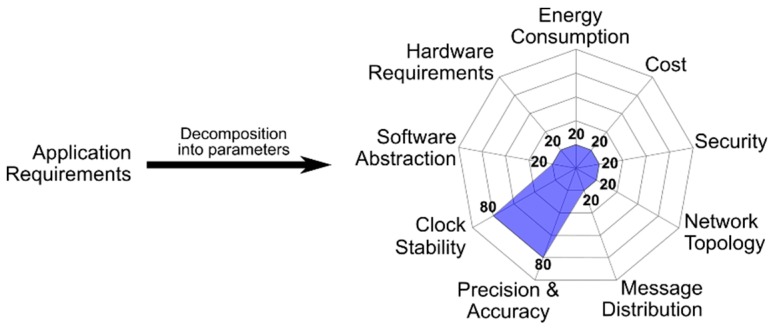
Graphical representation of the application requirements. This chart is generated with the answers of the Internet of Things (IoT) developers through the input web form.

Figure 4 shows the graphical representation of an example of application requirements. In this case, the application has high requirements in clock stability, precision and accuracy. However, the rest of the parameters have few constraints.

The input forms offered to the developers asking for the time-synchronization requirements of their IoT application are shown in the following subsections.

### 4.1. Synchronization Objective

The synchronization strategies are very different according to their purpose. Although an improvement in hardware means that all time measures improve, software solutions are very dependent on the objective of the synchronization protocol. We differentiate three types of objectives: MAC schemes, cooperative transmission and data fusion. The MAC schemes synchronization strategies pursue the objective of synchronizing the wireless IoT network in the MAC layer. In this way all the communication protocols can use differentiated time slots to transmit packets and avoid collisions. The cooperative transmission applications synchronize their communications to achieve collective goals, for example, to make constructive interference. The data fusion applications need synchronization to merge all the data collected for the individual nodes to compound a general picture of the whole network. In this case there is a radio button selection tool where only one objective can be selected.

### 4.2. Energy Consumption and Cost

Regarding energy consumption, if in the implementation of the node’s software there are transitions in the microcontroller state between active mode and low-power mode, it is necessary to take into account the clocks that can remain active in low-power mode and their accuracy. The same is applicable to the transitions between active mode and sleep mode in the radio transceiver. Those transitions can affect the synchronization strategy to follow. If developers are looking for a solution with limitations in energy consumption, or the energy consumption should be as small as possible, only certain synchronization strategies will be viable. If enough energy consumption data is supplied to the system, an estimation can be given according to the percentage of time that the system spends in each state. In that case the methodology has to evaluate if doing more operations locally and sending fewer wireless messages is worth it, or if it should send more messages and perform fewer operations.

In a similar way, if the overall cost of the synchronization strategy is a factor to take into account, some solutions would be non-viable or not recommended. For example, the use of GPS modules or some expensive temperature-compensated crystal oscillators (TXCO).

### 4.3. Scenario and Security Analysis

The network size is the number of nodes that compose the network. There are many factors such as the network topology, the message distribution scheme, or the difficulty of synchronizing many or just a few nodes in the network, among others, that depend on the network size. The physical localization where the IoT network is deployed determines which synchronization strategies are more adequate in each case. So, in this case the question is: what is the total number of nodes in the network?

The synchronization scope is the range of action where the time synchronization must be effective. If the time-synchronization strategy needs to synchronize all the nodes in the network or only some of them is an important parameter to know for the tools that execute the methodology. In addition, sometimes, the IoT network needs to be synchronized with third-party references, such as coordinated universal time (UTC).

Knowing the maximum and minimum physical distance that exists between the nodes of the network allows this tool to deduce other parameters. In this way the methodology can estimate the radio transmission power and, therefore, the energy consumption of wireless communications. This parameter also allows the tools that execute the methodology to calculate the one-way communication time. With it, the maximum and minimum communication time could be estimated and therefore the propagation time that affects the time synchronization over the network.

The security of the time-synchronization strategy indicates how critical is to assure the authenticity of the time source. There are some applications that require a secure and reliable time-synchronization scheme for the IoT network because they are placed in hostile environments. Those time-synchronization protocols incorporate special strategies to fight against fake timestamp broadcasters or they have some prevention strategies against malicious attacks.

The topology of the network is also related to the previous concept of the distance between nodes. There are some synchronization protocols that make use of specific network topologies, such as linear, star, tree or mesh. The message distribution scheme is also very dependent on the network topology. Message distribution is usually critical in the synchronization protocols, since it is through this distribution of messages that deterministic and non-deterministic pathways appear. That is why many protocols use very specific message distributions.

Communications in the network can be multi-hop, meaning that the messages can go through the network further than the physical distance between two adjacent nodes. If the network allows multi-hop communication, the communication distance can cover the whole network. If the input form is filled with this communication distance and also knows the physical distance between nodes, it is possible to infer whether single hop or multi-hop communications are necessary. This way the tool can discard some network topologies and some message distribution systems. The question in this case is: does your network need multi-hop communications?

Another way to get relations and to make estimations is the fact that knowing the number of nodes in the network in combination with the span of communications and the space between nodes, the tool can get an idea of the topology of the network. Also, according to the number of nodes, the clock dispersion and therefore the error of the time-synchronization strategies varies.

### 4.4. Application Requirements

As commented in previous sections, every application has particular requirements. The triangle of relationships between the maximum error allowed by the application, the accuracy of the clock and the period between synchronization rounds offers the tool information of vital importance when selecting time-synchronization strategies. Different questions are raised in this section, such as: what is the maximum error allowed for time synchronization?

Another useful detail to know is if the local clocks in the nodes are corrected continuously or if they are allowed to run freely (without local corrections). Furthermore, if the clocks are corrected continuously, this correction operation can be done on demand when an event is detected, or it can be done periodically at intervals.

Another important topic is whether the synchronization in the network is done exceptionally or if the time-synchronization strategy needs to have the clocks synchronized throughout the lifetime of the application. This affects the tasks and the percentage of execution time in the microprocessor used by the synchronization strategy. If continuous local corrections are made by the nodes, it may be necessary to do skew calculations, that is, the estimation of the deviation of their local clocks from the reference one. These mathematical calculations may require more complex operations, operations with floating point, or they may need to store more variables in memory. It may also occur that our hardware platform provides us with some tools that facilitate calculations.

The tool needs to know if the reference clock is static or not, that is, if it always comes from the same source, or if it is a dynamic reference.

The convergence time of the synchronization strategy is another factor to consider. The greater the time of convergence, the greater the percentage of central processing unit (CPU) that is dedicated. In addition, the longer it takes to synchronize the network, the longer it takes to perform the synchronized tasks required by the application.

### 4.5. Microcontroller Requirements

A wireless IoT end device can have several clocks. The tool makes better decisions if it knows the number, type, frequency and accuracy of all the clock sources used. Having one or several clock sources allows the tool that executes the methodology to select mixed techniques, such as the virtual high-resolution timer (VHT) [29], or switch between the working states of the microcontroller by turning off some clocks. If the tool knows the typology and frequency of the clock sources, it can establish whether the clocks are affected by environmental factors or not. The tool can also estimate the range of accuracy of a clock only by knowing its type. The tool knows that internal relaxation clock sources have poor accuracy and that they are very dependent on factors such as the temperature. Clock sources based on crystal oscillators have good accuracy and good precision. The accuracy of the clocks is one of the parameters used to calculate the relationship between the maximum permissible error and the length of the intervals between rounds of time synchronization. Although the clock sources have a certain frequency, the system may work at a higher or lower frequency. The inverse of this frequency gives the tool what we refer to as bit time. The bit time sets some resolution limits. For example, the bit time for 32.768 kHz clocks is 30.5 us, but for an 8 MHz clock is 125 ns. In addition, the frequency can be used to estimate consumption if necessary.

The level of software abstraction is key for choosing an adequate synchronization strategy. If the software architecture is based on an operating system or if nodes work on bare machines, this leads the tool to certain solutions. An operating system can facilitate the tasks of integration or implementation of the software, but at the same time the operating system could mask many tasks and executions. In a bare machine system, it is more difficult to implement software but there is more control over the execution of tasks and the code is better at dealing with the hardware constraints. A system with an operating system is slower when executing code because there is code overload due to the context changes, so there is a trade-off between low-level control and efficiency in code development.

Finally, regarding the hardware platform, knowing the memory size is interesting to select the type of strategy to use. It is necessary to consider both types of memory, the size of the flash memory and the size of the random-access memory (RAM). Flash memory affects the size of code to be implemented, while RAM memory affects the amount of data that can be stored in execution time. In the case of some synchronization strategies it could affect the number of offset timestamps that can be stored for skew calculations using methods such as least squares regression [23,30].

### 4.6. Transceiver Requirements

The frequency band of the transceiver fixes some characteristics such as the wavelength and therefore the transmission range. Taking into account the frequency band, an estimation of the one-hop communication span can be made. To estimate that communication span it is also necessary to know the type of antenna (strip, external, ceramic, etc.), and the configured transmit power. An estimation of the scope of communications can be made with these data. The baud rate and other details about the modulation are asked in this input form. The type of modulation to be used for communications is also a factor that can filter the type of synchronization strategy to follow. For example, to use inherent synchronization protocols, such as Glossy, a minimum-shift keying (MSK) modulation is needed [31].

### 4.7. Report Results

The tool that executes the methodology considers that if there are no limitations the best resources can be used giving priority to the accuracy in time synchronization. For example, if there are no hardware limitations, the methodology can choose the hardware platform and transceiver that gives the best results without considering their energy consumption or price.

The tool always generates a result. In some cases, depending on the requirements of the application and the decisions of the developer, a group of synchronization strategies is proposed as a solution. In other cases, when there is a harder dependency between the parameters and the degrees of freedom are reduced, the result of unfeasibility can be reached. In this case, the result is accompanied by the causes and trade-offs that make it unviable.

There are dependencies among all the parameters evaluated by the tool so that, sometimes, when trying to maximize some of them, others are minimized. This means that many of the questions are related to each other or their response may affect more than one parameter.

**Figure 5:**
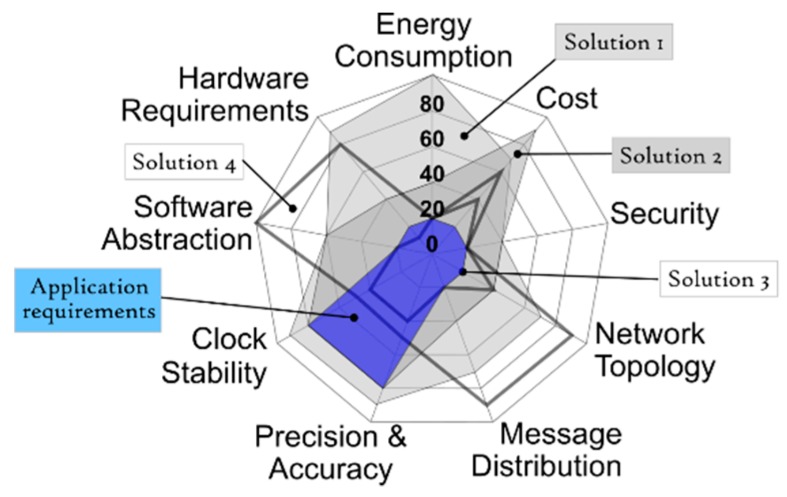
Graphical representation of how a valid solution is selected. Here the application requirements shape (blue area) is superimposed over all the available solutions in the database. Only the solutions 1 and 2 are valid (grey area) because they fully contain the area of this specific application requirements.

All the possible solutions, hardware and/or software, fulfill a series of trade-offs between the requirements. With all those trade-offs, a radar chart is formed that graphically shows the dependency between the parameters and the quantitative value of each parameter. Figure 5 shows a graphical representation of how a solution is elected. Figure 5 is a special figure, just for this article, to clarify how the tool filters the different available solutions. The application requirements (Figure 4) are superimposed over the available solutions in the database (Figure 2) giving Figure 5 as a result. As can be seen, only the solutions 1 and 2 fully contain the area of the application requirements, therefore, only solutions 1 and 2 are valid in this example case. The radar chart offers a visual way to know, through areas, which solutions are valid and which are not. It can be seen that the smaller the area of the radar chart, the less restrictive or the greater the freedom when choosing a synchronization strategy. In contrast, the greater the area of the radar chart, the more selective and demanding are the requirements to choose a valid synchronization strategy. All the viable solutions that pass the filter are included in the chart. Any modification in the input blocks causes a modification in the requirements or on the freedom to choose an appropriate strategy, and therefore the tool generates a different radar chart.

As mentioned, all the time-synchronization strategies have previously been included in a database with a value assigned to each of their parameters. In this way all the synchronization strategies are parametrized and can be represented on a radar chart. This way the radar chart offered to the developers is generated thanks to the information collected through the input forms.

Once the radar chart has been generated with all possible solutions, there are several possibilities before finalizing the methodological cycle.

- The developers can directly select one of the possible solutions offered by the tool.
- Under certain circumstances, an automatic selection can be made by the tool based on certain parameters. The solution with the best precision and accuracy and bigger area is the one offered as the best automatic solution.
- When the time-synchronization strategies are inserted in the database, some of them have an associated pseudocode. If any of them are included in the set of available solutions, the pseudocode is also offered to the developers to facilitate the implementation task.

## 5. Use Cases

In this section we propose two use cases: one of them with a large freedom to make decisions and a much more restrictive one. In this way we show how the methodology works in different circumstances and applications giving an appropriate solution in every case.

### 5.1. Environmental Monitoring Application

In this first use case, we face the selection of a synchronization strategy with a large freedom for decisions. Multiple details of the web form of inputs are irrelevant because of that lack of restrictions.

#### 5.1.1. Assumptions

In this case the use of the methodology pursues the goal of finding a synchronization mechanism for an environmental monitoring application. The application monitors multiple parameters, such as temperature or humidity, covering a large area of terrain using a wireless IoT network. It is necessary to aggregate all the data in order to have a global vision of how the monitored variables in the area evolve. The aggregated data must be time synchronized to obtain an accurate picture of the environment. The information must be transmitted periodically and continuously to a processing center. As the parameters under monitoring vary slowly with time, their acquisition is done every 10 min.

#### 5.1.2. Methodology

Regarding the first input block, in this case the objective of the synchronization strategy is the data fusion. The network size is imposed by the size of the area under monitoring and the localization is mainly outdoors. There is a special interest in the energy consumption (value of 80) because the IoT end devices are scattered throughout an extensive area and they are battery-powered. The cost (value of 50) is interesting because in this case the number of end devices is large. The application is developed in a in a barely hostile environment and therefore it has no special security requirements (value of 5). Neither the topology, nor the distribution of messages (value of 50) are very demanding in their requirements. Several options like star or mesh topologies are valid. The message distribution only needs multi-hop communications. The application requires a good clock stability (value of 80) but it does not need very good precision or accuracy (value of 40) because the environmental changes are slow and the sample period is adjusted to the application. Finally, there is almost full freedom (value of 10) to choose the hardware platform and also the software.

**Figure 6:**
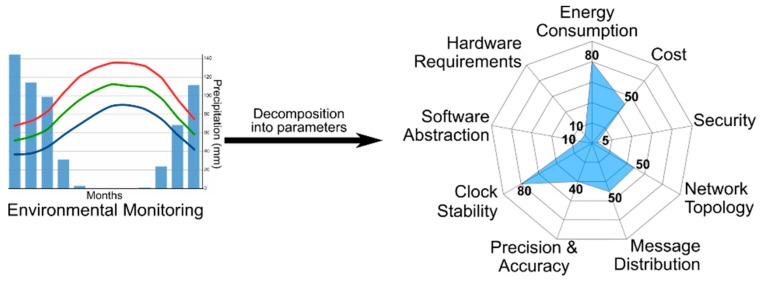
Radar chart generated for the specific requirements of an environmental monitoring application. Low values represent few constraints or big freedom in the selection, and large values represent high demands or very restrictive conditions.

Given the premises, and following the methodology, the tool generates a radar chart with a very narrow area (see Figure 6), meaning that many of the solutions with the data fusion objective are valid. From all the possible solutions offered by the radar chart, there is one best solution that the tool selects automatically. The best option proposed by the tool is the use of RTC modules based on TXCO in each of the nodes. The recommended transceiver uses sub-gigahertz communications because the distances between nodes can be large and because the data transferred from one node to another is small. The network topology recommended is a mesh topology because it is the most adaptable to the scenario. The RTCs are corrected on demand with reference timestamps reducing in this way the message exchange due to time synchronization.

### 5.2. Structural Health Monitoring Application

In this second case, following the methodology the tool needs to find a viable solution for a more demanding application regarding time synchronization. In this case, most of the input forms are filled with details because the requirements are more demanding.

#### 5.2.1. Assumptions

The tool faces an application for structural health monitoring. The time-synchronization requirement in these applications is stricter and requires a maximum deviation between the clocks in the network of 1 millisecond [32,33]. However, the more precise it is, the better the results. In these applications the main task is the acquisition of accelerations and, therefore, the task of synchronization, cannot, under any circumstances, interrupt the acquisition.

The system has to be portable to be installed in different structures and it should be battery powered to allow its deployment in different scenarios. In addition, in this case, we assume that a hardware and software platform are already defined. Nodes are based on the STM32L4 Nucleo platform with a Spirit1 transceiver, with the YetiOS operating system [34], a mesh network topology and multi-hop message distribution.

#### 5.2.2. Methodology

In this case the objective is the same as in the previous use case, the data fusion, but with a much more demanding synchronization accuracy. The network size and the localization of the scenario are variable. The energy consumption is a parameter (value of 40) that has relative importance. The wireless IoT end devices are battery-powered but they can be charged every day. The cost (value of 40) depends only on the number of end devices needed for the specific scenario. There are no special requirements regarding security because this is a mobile system and it is considered that no particular attacks are going to occur (value 10). The network topology and the message distribution (value of 60) are previously defined. For this application the time precision and accuracy (value of 90) are very important for identifying the modal modes of the structures. A clock with good stability (value of 80) is also important to facilitate the clock synchronization operations. The synchronization messages have to be timestamped in the MAC layer and that is the reason for a value of 60 in the abstraction layer. Finally, since the hardware platform is also previously defined, the requirement is high (value of 80).

From the introduced data, the tool knows the number, type, frequency and accuracy of the clock sources. YetiOS makes transitions between active and low-power modes always keeping the internal RTC working, but there are some clocks that go into low-power mode. The tool also knows all the information relative to the radio transceiver. Making use of the decision filters, the tool knows that to keep the time deviation below 1 millisecond, making use of a 20 ppm accuracy clock, the synchronization rounds should be undertaken every 50 s or less. As the structure to be monitored can be outdoors, the tool takes the temperature into account. Considering a bridge whose surface could be at 50 °C, the 20 ppm accuracy clock can have an error up to 40 ppm, so the synchronization rounds should be reduced to 25 s. Given that the hardware platform is able to do the relevant calculations, if the synchronization strategy chooses to make internal corrections to the clock, it could calculate the skew and therefore correct the offset periodically.

**Figure 7:**
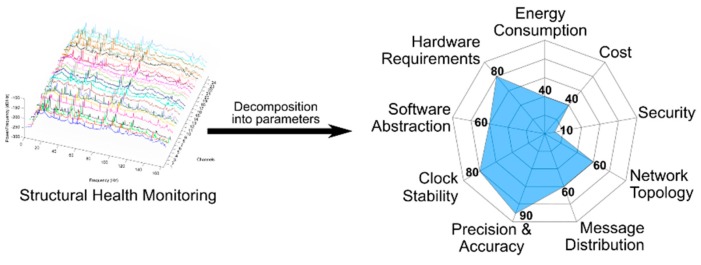
Radar chart generated based on the input form for a specific structural health monitoring application. Since the area of the chart is big, it represents that the application is very demanding in the considered parameters.

In this case the radar chart generated by the methodology is shown in Figure 7. The area in this case is big reflecting the very demanding requirements. Since the area is large, only a few synchronization strategies fully contain it, meaning that only a few strategies meet the requirements.

The hardware platform used has the computing capacity necessary to carry out the necessary calculations in most of the protocols that exist to date. As a result, those synchronization protocols that allow working in a mesh network and whose accumulated multi-hop error is kept below 1 millisecond would be offered, such as Glossy [31].

## 6. Conclusions

Time synchronization in wireless IoT networks is a key element for the proper operation of many applications. Each application has different requirements related to time synchronization. That is why many synchronization strategies have been developed and published over the years. When IoT application developers need a synchronization strategy they must face the difficulty of this selection.

This selection must take into account many parameters, and, in many cases, requires prior knowledge in the subject of synchronization in wireless IoT networks. Given this difficulty, we have detected the need to develop a methodology to facilitate the task of selecting a synchronization strategy adapted to the needs of each IoT application.

With this methodology the IoT application developers focus on the problem of developing the necessary services of the application, abstracting them from the problem of choosing a synchronization strategy. Using an analogous example, if developers want to have a secure application, developers do not need to be security experts if they have a methodology that indicates the steps and techniques to be applied.

In this article, a methodology for choosing time-synchronization strategies for wireless IoT networks is presented. A tool is also presented to execute this methodology. Making use of this tool, developers can introduce the requirements of their IoT applications, and a solution adapted to those requirements is generated. The methodology presented in this work is repeatable, reproducible, and allows developers to find synchronization solutions that fit their needs and those of the application.

The tool presented makes use of a database of time-synchronization strategies. In this way the tool is expandable to new solutions that appear in the future and, therefore, can be enriched with new options and alternatives.

The combination of the methodology and the tool creates an abstraction layer to the developers of IoT applications with respect to the time-synchronization strategies. IoT application developers do not need to be experts in the field of time synchronization to select the appropriate solution for their specific case. Experts and non-experts in the field get the same technical solution making use of the methodology cycle. This is possible because all the possible time-synchronization strategies have previously been parametrized and inserted into the tool database by experts.

The main advantages of applying this methodology to wireless IoT networks are:

- It generates a rapid response regarding viability. As the methodology is based on a series of steps, and the viability of a valid solution is evaluated in each step, it is not necessary to wait until the end of an implementation to know if it is possible to obtain a solution that fulfills the application requirements.
- It gives greater control to the developer. Developers participate actively in the different stages of the process, for example, introducing all the requirements and needs of the IoT application under development in the input forms of the tool.
- The tool used to execute the methodology establishes a relationship between the different parameters and their effect on synchronization. When prioritizing the parameters, the developers know which ones have more impact than others.
- It clarifies the relationship between the parameters of each synchronization strategy and the requirements of each application making use of graphical representations in the shape of a radar chart.

Two examples of use cases of the methodology and the tool have been given with two well differentiated scenarios with different characteristics and requirements. The behavior of the methodology has been shown in both cases giving valid results to the developer. This article demonstrates that the proposed methodology guides IoT application developers in the selection of a proper time-synchronization strategy that fulfills the requirements of their application.
